# MRI Based Radiomics Compared With the PI-RADS V2.1 in the Prediction of Clinically Significant Prostate Cancer: Biparametric vs Multiparametric MRI

**DOI:** 10.3389/fonc.2021.792456

**Published:** 2022-01-20

**Authors:** Tong Chen, Zhiyuan Zhang, Shuangxiu Tan, Yueyue Zhang, Chaogang Wei, Shan Wang, Wenlu Zhao, Xusheng Qian, Zhiyong Zhou, Junkang Shen, Yakang Dai, Jisu Hu

**Affiliations:** ^1^ Department of Radiology, The Second Affiliated Hospital of Soochow University, Suzhou, China; ^2^ School of Medical Imaging, Biomedical Engineering, Xuzhou Medical University, Xuzhou, China; ^3^ Department of Ultrasound, Nanjing Drum Tower Hospital, Nanjing Medical School, Nanjing, China; ^4^ Department of Radiology, Jiangsu Jiangyin People’s Hospital, Jiangyin, China; ^5^ Suzhou Institute of Biomedical Engineering and Technology, Chinese Academy of Sciences, Suzhou, China; ^6^ School of Biomedical Engineering (Suzhou), Division of Life Sciences and Medicine, University of Science and Technology of China, Suzhou, China; ^7^ Institute of Imaging Medicine, Soochow University, Suzhou, China

**Keywords:** prostate cancer, radiomics, biparametric MRI, multiparametric MRI, PI-RADS v2.1

## Abstract

**Purpose:**

To compare the performance of radiomics to that of the Prostate Imaging Reporting and Data System (PI-RADS) v2.1 scoring system in the detection of clinically significant prostate cancer (csPCa) based on biparametric magnetic resonance imaging (bpMRI) vs. multiparametric MRI (mpMRI).

**Methods:**

A total of 204 patients with pathological results were enrolled between January 2018 and December 2019, with 142 patients in the training cohort and 62 patients in the testing cohort. The radiomics model was compared with the PI-RADS v2.1 for the diagnosis of csPCa based on bpMRI and mpMRI by using receiver operating characteristic (ROC) curve analysis.

**Results:**

The radiomics model based on bpMRI and mpMRI signatures showed high predictive efficiency but with no significant differences (AUC = 0.975 vs 0.981, p=0.687 in the training cohort, and 0.953 vs 0.968, p=0.287 in the testing cohort, respectively). In addition, the radiomics model outperformed the PI-RADS v2.1 in the diagnosis of csPCa regardless of whether bpMRI (AUC = 0.975 vs. 0.871, p= 0.030 for the training cohort and AUC = 0.953 vs. 0.853, P = 0.024 for the testing cohort) or mpMRI (AUC = 0.981 vs. 0.880, p= 0.030 for the training cohort and AUC = 0.968 vs. 0.863, P = 0.016 for the testing cohort) was incorporated.

**Conclusions:**

Our study suggests the performance of bpMRI- and mpMRI-based radiomics models show no significant difference, which indicates that omitting DCE imaging in radiomics can simplify the process of analysis. Adding radiomics to PI-RADS v2.1 may improve the performance to predict csPCa.

## Introduction

Prostate cancer (PCa) is among the most common malignancies in the male population worldwide, and its incidence rate is rapidly increasing in China, which has become an important risk factor affecting the health of the elderly (1, 2). The early and accurate detection and diagnosis of PCa, especially clinically significant PCa (csPCa), are unequivocally of great significance for patients and have an important impact on their therapeutic response and prognosis.

In recent years, magnetic resonance imaging (MRI) has generally been considered to be the most reliable noninvasive imaging technology for the detection and localization of PCa (3). Updated in 2015, the Prostate Imaging Reporting and Data System version 2 (PI-RADS v2) was released to standardize of multiparametric MRI (mpMRI), including T2-weighted imaging(T2WI), diffusion-weighted imaging (DWI), and dynamic contrast-enhanced (DCE) imaging (4). In 2019, the PI-RADS version 2.1 was newly described, and a simplified biparametric MRI (bpMRI) comprising T2WI and DWI was proposed (5). To date, many studies have demonstrated that the use of bpMRI protocols don’t significantly reduce PCa detection rates and is comparable to mpMRI protocols (6–8). Meanwhile, bpMR protocols also have several advantages, such as a shorter examination time, reducing cost and avoiding potential adverse effects of gadolinium-based contrast agents. Nevertheless, given the growing employment of the PI-RADS, inter-reader variability remains an unavoidable problem. Thus, it is imperative to look for a quantitative diagnostic method to improve the performance of the PI-RADS for the early and accurate diagnosis of PCa.

Radiomics research extracts high-throughput and quantitative features from multi-modal medical images and converts them into high-dimensional mineable information related to tumour pathophysiology using machine learning algorithms, and these features might aid in clinical diagnosis and decision-making (9–11). At present, manual delineation of the region of interest (ROI) is still the main method of radiomics analysis, but it inevitably consumes a certain number of human resources and time costs. Although many researches have confirmed that the accuracy of bpMRI in detecting PCa is comparable to that of mpMRI, but the diagnostic abilities of bpMRI- and mpMRI-based radiomics for PCa, especially in csPCa, have not been compared.

Therefore, our study aimed to (1) compare the performance of radiomics based on bpMRI and mpMRI in the prediction of csPCa and (2) to explore whether radiomics can enhance the performance of the PI-RADS v2.1 in the diagnosis of csPCa.

## Materials and Methods

### Patient Cohort

**Figure 1:**
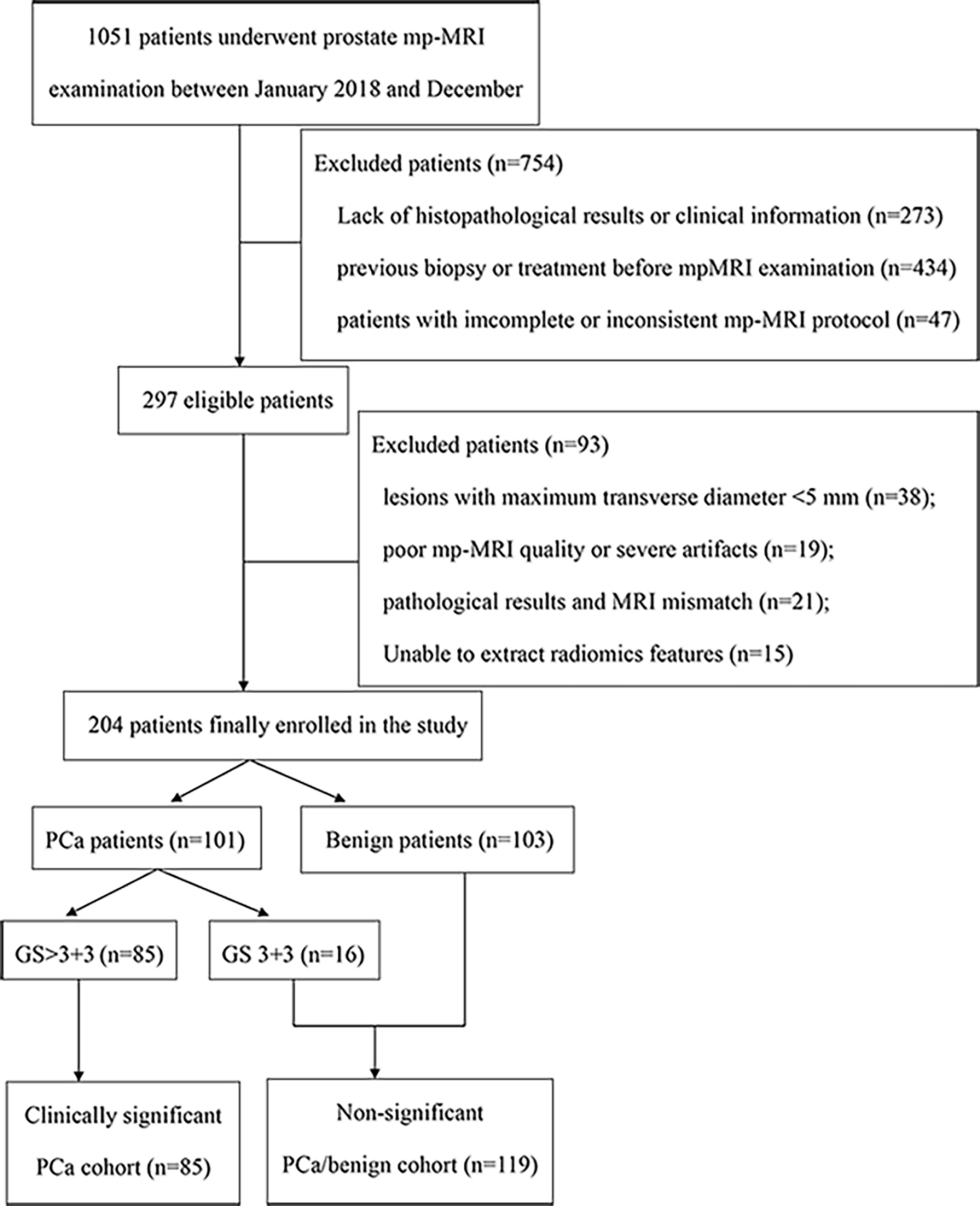
Flow chart of patients’ recruitment pathway.

This retrospective study was approved by the institutional ethics committee of our hospital. Between January 2018 and December 2019, a total of 1051 consecutive patients who underwent a 3.0 T prostate mpMRI examination were recruited. The inclusion criteria were as follows: (1) patients with clinical symptoms or elevated PSA levels with a suspicion of PCa; (2) patients who underwent a prostate 3.0 T mpMRI examination; (3) transrectal ultrasound (TRUS)-guided prostate biopsy/MRI-TRUS fusion targeted biopsy or radical prostatectomy with confirmed pathological results after MRI examinations; and (4) no prior prostate endocrine therapy, biopsy, surgery, or radiation therapy before the MRI examination. The exclusion criteria were as follows: (1) lesions with a maximum transverse diameter <5 mm; (2) poor mpMRI quality or severe imaging artefacts; and (3) pathology yielded prompted lesions that were difficult to delineate on MRI (the lesion location could not be determined on MRI). Details of the patient recruitment pathway are shown in **Figure 1**. Finally, we enrolled 204 patients: 101 patients with PCa and 103 patients without any histological evidence of cancer. The patients were randomly divided into two groups (the training and testing cohorts) at a ratio of 7:3.

### Magnetic Resonance Imaging Protocols

All patients were scanned with a 3.0 T MRI scanner (Philips Intera Achieva, Best, Netherlands) with a 32-channel body phased array coil as the receiving coil. The scan sequences included sagittal T2WI, axial T2WI, T1WI, DWI (b values of 0, 100, 1000 and 2000 sec/mm2) and DCE. Apparent diffusion coefficient (ADC) maps were calculated on a designated workstation with b values of 100 and 1000 sec/mm2. For the DCE images, gadolinium chelate, at a dose of 0.1 mmol/kg of body weight, was injected intravenously at a rate of 2.5 mL/s, with a temporal resolution of 5.8 sec/phase. Before the DCE scan, T1 mapping scans were performed at three reversal angles: 5°, 10° and 15°. Details of the protocol, including the sequence type, repetition time/echo time (TR/TE), section thickness, field of view (FOV), and matrix, are summarized in **Table 1**.

**Table 1:** Prostate mp-MRI protocol.

Sequence	TR (ms)	TE (ms)	FOV (mm×mm)	Slice thickness (mm)	Slice gap (mm)	Matrix	NEX
T2WI-axial	3000	100	220×220	3.0	0.00	276×278	3
T2WI-sagittal	4978	100	240×180	1.5	0.15	240×161	2
DWI-axial*	6000	77	260×260	3.0	0.00	104×125	2
T1WI-axial	556	8	249×415	5.0	0.00	276×406	1
DCE	3.2	1.5	220×220	3.0	0.00	124×121	2

TR, repetition time; TE, echo time; FOV, field of view; NEX, number of excitations.

*b=0, 100, 1000, 2000 s/mm^2^.

### Reference Standard for Pathology

All patients underwent TRUS-guided 12-core systematic biopsy, besides, MRI-TRUS fusion targeted biopsy was used for suspicious PCa lesions on MRI (with PI-RADS ≥ 3) and 2–3 targeted cores were added for these lesions. An ESAOTE Mylab Twice color Doppler ultrasound device equipped with a real-time virtual sonography (RVS) imaging fusion system was used for MRI-TRUS fusion targeted biopsy. The ultrasonic probe model was an EC-123,7.5- MHz transrectal end-fire probe (EsaoteSpA, Genova, Italy). Partial of the patients underwent radical prostatectomy after biopsy. The biopsy procedure was performed by a senior urologist with over 5 years of experience. The histopathological specimens were assessed by experienced pathologists in our hospital according to the International Society of Urological Pathology (ISUP) 2014 updated Gleason score grading system.

### PI-RADS Evaluation

The MR images were retrospectively reviewed by two radiologists with more than 5 years of experience in the diagnosis of PCa. The readers were blinded to all clinicopathological information. According to the PI-RADS v2.1 protocol, two readers interpreted the same set of MRI images at two different times to determine the PI-RADS categories derived from the index lesion of each patient (the index lesion was marked and recorded by the radiologist who delineated the ROI): (a) first with the bpMRI protocol (T2WI and DWI), and (b) second with the mpMRI protocol (T2WI, DWI and DCE) after four weeks. If there was any disagreement between the two readers, they reached a consensus by discussion and determined the final PI-RADS categories.

### PCa Lesion Segmentation on T2WI and ADC Maps

All images were normalized before feature extraction. The images were normalized with the average of the standard deviations as the center, and the normalize scale was set to 100. The B-spline interpolation method was used to recompress all voxels of the image to a voxel spacing of millimetres, and the voxel array shift and bin width were set to 300 and 5, respectively, to ensure that each voxel was positive.

For consistency between ROIs delineated in both T2WI and ADC images, a radiologist together with a pathologist depicted all the ROIs complied with the following criteria with ITK-SNAP (http://www.itksnap.org/pmwiki/pmwiki.php). First, according to the detailed records of the prostate biopsy (including the injection site and depth) or pathological results of radical prostatectomy, the location of the lesion was determined; then, the corresponding lesion was matched on MR images in accordance with the location described by pathology. The radiologist manually delineated the ROIs slice by slice along the lesion boundary, obtaining a volume of interest (VOI). Referencing the PI-RADS V2.1 scoring criteria, the range of lesions was determined. Lesions in the peripheral zone (PZ) were mainly based on the ADC imaging (supplemented with DWI and DCE imaging), while lesions in the transitional zone (TZ) were mainly based on T2WI (supplemented with DWI and the ADC imaging). During the process of sketching the ROIs, the urethra, ejaculation tube, and seminal vesicle root structure were avoided. For a patient with multiple lesions, the index lesion was determined as the highest region of GS that was confirmed by biopsy/pathology or the maximum diameter of the lesion if the GSs were the same.

### PCa Lesion Segmentation on DCE Imaging

**Figure 2:**
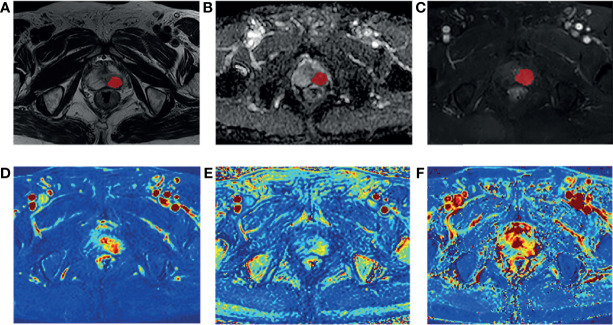
A 80-year-old man diagnosed with csPCa in TZ (PSA,59.90 ng/mL; biopsy GS, 3 + 4 = 7). Example segmentations (red masks) of the tumor overlaid on on axial T2-weighted imaging (T2WI) **(A)**, apparent diffusion coefficient (ADC) map **(B)**, and dynamic contrast-enhanced MRI (DCE-MRI) **(C)**. The ROIs were selected based on the enhanced T1WI and then matched to Ktrans map **(D)**, Kep map **(E)**, and Ve map **(F)**.

The T1 mapping and DCE-MRI data were transferred into the Omni-Kinetic software (GE Healthcare, China) to obtain the perfusion and permeability parameters of the lesions. The extended tofts linear model and the population arterial input function (AIF) embedded in the software were used for analysis, and the following quantitative parameters maps were obtained automatically: Ktrans, Kep, and Ve. For patients with positive DCE enhancement, a series of ROIs of lesions were manually delineated slice by slice using ITK-SNAP on the eighth dynamic contrast images. The eighth contrast images were chosen because that the lesions showed significantly enhancement while the entire prostate background was not enhanced at this stage. While for patients with negative DCE enhancement, it is necessary to refer to the T2WI and DWI/ADC sequence to locate the lesion on the enhancement images (**Figure 2**). The sketched ROIs were then matched to the pharmacokinetic maps.

### Feature Extraction and Selection

Radiomics features of the lesions were extracted using PyRadiomics (version 2.1.0; https://pyradiomics.readthedocs.io/en/2.1.0/), which is an open-source function package for extracting radiological features from medical images. Seven types of radiomics features were derived from each mpMRI sequence, including first-order statistics features, shape‐based features, grey level cooccurrence matrix (GLCM) features, grey level run length matrix (GLRLM) features, grey level size zone matrix (GLSZM) features, neighbouring grey tone difference matrix (NGTDM) features, and grey level dependence matrix (GLDM) features.

To ensure the stability of the features, two radiologists drew the ROIs slice by slice independently on the MR images of 40 patients. Only the features with inter- and intra-class correlation coefficients (ICCs) > 0.75 were included in the following analysis.

To eliminate the differences in the value scales of the radiomics features, all of the features were normalized to zero mean and unit variance by a standardized method before feature selection. The t-test algorithm was used to filter the extracted features, and then, the least absolute shrinkage and selection operator (LASSO) regression method was applied to select the optimized subset of features with the optimal tuning parameter (λ). Ten-fold cross-validations were used to selected the optimal models.

### Model Construction and Statistical Analysis

Two radiomics models were established on training data with a support vector machine (SVM) classifier to distinguish csPCa from non-csPCa/benign lesions based on bpMRI radiomics features and mpMRI radiomics features, respectively.

All statistical analyses were performed using R software (version 4.1.0, http://www.Rproject.org), Statistic Package for Social Science (SPSS, version 21.0, https://www.ibm.com/cn-zh/analytics/spss-statistics-software), GraphPad Prism (version 9.0, https://www.graphpad.com/scientific-software/prism/) and MedCalc (version 15.8, https://www.medcalc.org). Continuous variables conforming to a normal distribution are expressed as the means ± SDs and ranges, and an independent sample T-test was used for analysis. Qualitative variables and continuous variables with a non-normal distribution are expressed as the medians (lower quartile, upper quartile), and were analysed using the Mann-Whitney U test. The inter-observer agreement of the PI-RADS v2.1 score was evaluated by weighted Cohen’s kappa statistics.

The testing data were used to verify the diagnostic efficacy of the predictive models, and the differences between the radiomics models vs. the PI-RADS v2.1 score based on bpMRI and mpMRI in identifying csPCa and non-csPCa/benign lesions were analysed with receiver operating characteristic (ROC) curves. The DeLong test was used to compare the significant differences in terms of the AUC values. P < 0.05 was considered statistically significant.

## Results

### Patient Characteristics

A total of 204 patients were ultimately enrolled in our study, in which 85 (42%) patients had csPCa and 119 (58%) patients had non-csPCa/benign lesions [78 prostatic hyperplasia (BPH), 25 prostatitis and 16 low-grade PCa with GS 3 + 3]. 50/204 (25%) patients underwent radical prostatectomy and the prostatectomy specimen results were used as the reference standard, while the remaining 154/204 (75%) patients who had only undergone systemic biopsy/MRI-TRUS fusion targeted biopsy took the biopsy specimen results as the standard. After randomly dividing the patients at a 7:3 ratio, 142 patients were allocated to the training cohort and 62 patients were allocated to the testing cohort. The demographic data of the enrolled patients are shown in **Table 2**.

**Table 2:** Patient characteristics.

Characteristics	csPCa (n = 85)	Non-csPCa/benign lesions (n = 119)	P value	Training cohort (n = 142)	Testing cohort (n = 62)	P value
Age(years)	72 (68-77)	70 (64-75)	0.027*	70 (64-76)	72 (67-77)	0.494
PSA (ng/ml)	31.99 (14.51-122.50)	10.34 (6.32-15.12)	0.000*	13.02 (8.35-30.50)	13.26 (7.30-37.47)	0.879
Location						
TZ	21	92		77	36	
PZ	41	27		48	20	
Both zones	23	0		17	6	
Gleason score						
GS 6	0	16		13	3	
GS 7	31	0		25	6	
GS 8	25	0		20	5	
GS 9	20	0		10	10	
GS 10	9	0		5	4	

csPCa, clinically significant prostate cancer; PZ, peripheral zone; TZ, transitional zone; PSA, prostate-specific antigen; GS, Gleason score.

*p < 0.05.

### PI-RADS v2.1 Score Based on bpMRI vs. mpMRI

**Figure 3:**
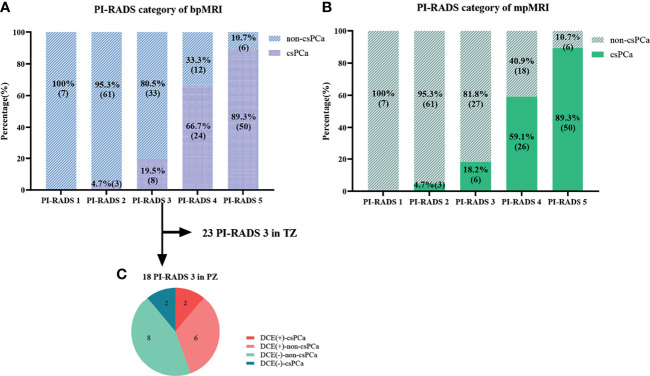
Comparison of the PI-RADS categories and pathological results. **(A, B)** Graphs show changes in the PI-RADS v2.1 category with bpMRI and mpMRI, as well as the relationship between the PI-RADS categories and pathological results. **(C)** Graph shows results of DCE and the histopathologic findings of the 18 patients with PI-RADS 3 lesions in the peripheral zone (PZ).

The category results of PI-RADS v2.1 with bpMRI and mpMRI were shown in **Figures 3** **A, B**. The weighted Cohen’s kappa value was 0.797 and 0.770 based on bpMRI and mpMRI images, respectively (P<0.001, **Table 3**), which demonstrated that there was substantial consistency between the two radiologists for PI-RADS v2.1 score. The inconsistency between bp-MRI and mp-MRI is mainly for lesions with PI-RADS 3 and PI-RADS 4. When using bpMRI, 18 patients with PI-RADS 3 had index lesions in the PZ, thus, DCE imaging was needed for these lesions using mpMRI. Among these patients, 8 patients with positive DCE findings were assigned PI-RADS 3 + 1. The csPCa detection rates in positive DCE findings and negative DCE findings were 25% and 20%, the total csPCa detection rate was 19.5% and 18.2% in PI-RADS 3 lesions with bpMRI and mpMRI, respectively (**Figure 3C**).

**Table 3:** Assessment of Interrater agreement for PI-RADS v2.1 score with bpMRI and mpMRI.

PI-RADS v2.1	R1 (bpMRI)				
R2 (bpMRI)	1	2	3	4	5
1	3	4	0	0	0
2	0	61	2	0	0
3	0	19	17	4	1
4	0	3	1	21	11
5	0	2	0	2	52
Kappa value	0.797				
P	<0.001*				
PI-RADS v2.1	R1 (mpMRI)				
R2 (mpMRI)	1	2	3	4	5
1	3	4	0	0	0
2	0	61	2	0	0
3	0	17	10	5	1
4	0	5	6	22	11
5	0	2	0	2	52
Kappa value	0.770				
P	<0.001*				

The degree of interrater agreement was interpreted by the kappa value as follows: 0.00-0.20 as no agreement to slight, 0.21-0.40 as fair, 0.41-0.60 as moderate, 0.61-0.80 as substantial, and 0.81-1.00 as perfect agreement. *P < 0.05 was considered statistically significant.

ROC curves were used to compare with bpMRI and mpMRI using the PI-RADS v2.1 to discriminate between patients with and without csPCa, as shown in **Figure 5**. In the training cohort, bpMRI and mpMRI yielded AUCs of 0.871 (95% CI: 0.780-0.962) and 0.880 (95% CI: 0.787-0.972), respectively. In the testing cohort, bpMRI and mpMRI yielded AUCs of 0.853 (95% CI: 0.760-0.946) and 0.863 (95% CI: 0.780-0.947), respectively. The sensitivity, specificity and accuracy of bpMRI and mpMRI in the detection of csPCa are reported in **Table 4**. The Delong test revealed no significant differences between the AUCs of bpMRI and mpMRI in either the training or testing cohort (p=0.888 and 0.868, respectively, shown in **Table 5**).

**Table 4:** ROC results of the PI-RADS, radiomics, and PI-RADS-radiomics combined models for predicting csPCa.

Model	PI-RADS				Radiomics				Combined model			
	bp-MRI		mp-MRI		bp-MRI		mp-MRI		bp-MRI		mp-MRI	
	Training	Testing	Training	Testing	Training	Testing	Training	Testing	Training	Testing	Training	Testing
AUC	0.871	0.853	0.880	0.863	0.975	0.953	0.981	0.968	0.982	0.969	0.986	0.977
SEN	0.800	0.760	0.817	0.760	0.950	0.880	0.950	0.920	0.967	0.960	0.967	0.960
SPC	0.890	0.892	0.890	0.919	0.963	0.973	0.988	0.973	0.976	0.946	0.988	0.973
ACC	0.852	0.826	0.859	0.855	0.958	0.935	0.972	0.952	0.972	0.952	0.979	0.968

AUC, Area under the curve; SEN, sensitivity; SPE, specificity; ACC, accuracy.

**Table 5:** Delong test of the PI-RADS, radiomics, and PI-RADS-radiomics combined models in the training cohort and testing cohort.

The Training Cohort	PI-RADS-bpMRI	PI-RADS-mpMRI	Radiomics-bpMRI	Radiomics-mpMRI	Combined-bpMRI	Combined-mpMRI
PI-RADS-bpMRI	–	0.888	0.030*	0.022*	0.017*	0.017*
PI-RADS-mpMRI	–	–	0.053	0.030*	0.033*	0.015*
Radiomics-bpMRI	–	–	–	0.687	0.664	0.505
Radiomics-mpMRI	–	–	–	–	0.940	0.703
Combined-bpMRI	–	–	–	–	–	0.743
Combined-mpMRI	–	–	–	–	–	–
The testing cohort	PI-RADS-bpMRI	PI-RADS-mpMRI	Radiomics-bpMRI	Radiomics-mpMRI	Combined-bpMRI	Combined-mpMRI
PI-RADS-bpMRI	–	0.868	0.024*	0.001*	0.007*	0.008*
PI-RADS-mpMRI	–	–	0.021*	0.016*	0.013*	0.006*
Radiomics-bpMRI	–	–	–	0.287	0.230	0.084
Radiomics-mpMRI	–	–	–	–	0.980	0.545
Combined-bpMRI	–	–	–	–	–	0.579
Combined-mpMRI	–	–	–	–	–	–

*P < 0.05 was considered statistically significant.

### Radiomics Model Based on bpMRI vs. mpMRI

**Figure 4:**
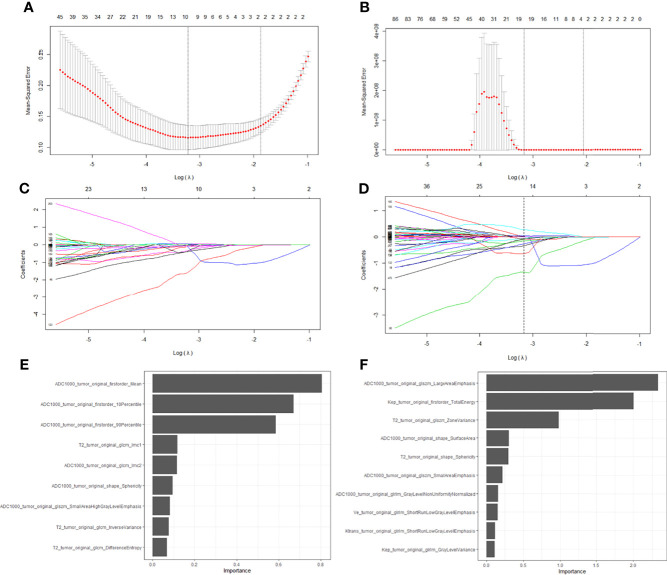
Feature selection with the LASSO regression method using bpMRI **(A, C)** and mpMRI **(B, D)** signatures. The importance of the selected top ten features in bpMRI and mpMRI images were shown in **(E, F)**, respectively.

A total of 535 features were extracted from T2W, ADC, and DCE images. After feature selection, nine features from bpMRI and fourteen features from mpMRI were used to develop the radiomics model. The LASSO feature selection process and the importance of the top ten selected features from bpMRI and mpMRI are shown in **Figure 4**.

**Figure 5:**
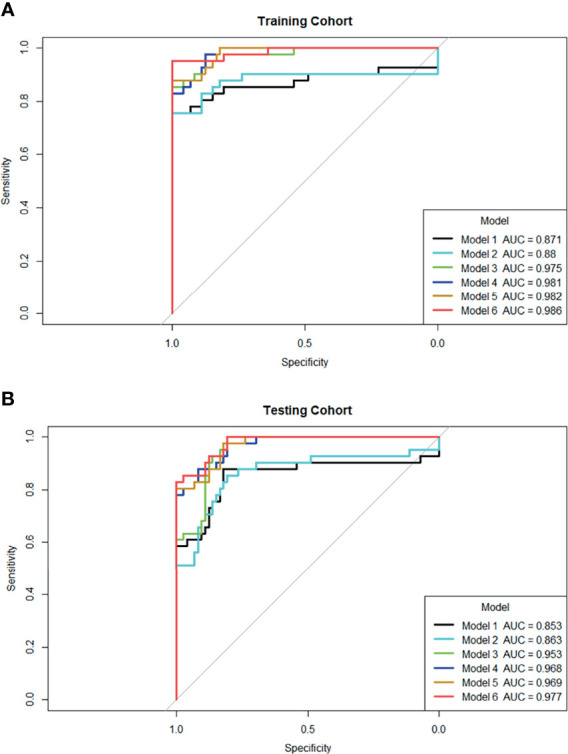
ROC curves for six models’ performance to distinguish csPCa in the training **(A)** and testing cohort **(B)**, respectively. Model 1: PI-RADS with bpMRI model; Model 2: PI-RADS with mpMRI model; Model 3: bpMRI-based radiomics model; Model 4: mpMRI-based radiomics model; Model 5: Combined PI-RADS and radiomics model with bpMRI; Model 6: Combined PI-RADS and radiomics model with mpMRI.

The radiomics model based on both the bpMRI and mpMRI signatures showed high predictive efficiency with AUC values of 0.975 (95% CI: 0.949-1.000) vs. 0.981 (95% CI: 0.964-0.999) in the training cohort and 0.953 (95% CI: 0.920-0.986) vs. 0.968 (95% CI: 0.942-0.995) in the testing cohort, as shown in **Figure 5**. However, the AUCs between bpMRI and mpMRI were nonsignificant in both the training and testing cohorts (p=0.687 and 0.287, respectively, shown in **Table 5**). The sensitivity, specificity and accuracy of the bpMRI- and mpMRI-based radiomics models in the detection of csPCa are reported in **Table 4**.

### Comparison Between the Radiomics Model and the PI-RADS v2.1

When using bpMRI, radiomics had a significantly higher AUC than the PI-RADS in both the training cohort [0.975 (95% CI: 0.949-1.000) vs. 0.871 (95% CI: 0.780-0.962), P = 0.030] and testing cohort [0.953 (95% CI: 0.920-0.986) vs. 0.853 (95% CI: 0.760-0.946), p= 0.024]. When using mpMRI, radiomics also had a significantly higher AUC than the PI-RADS in the training cohort [0.981 (95% CI: 0.964-0.999) vs. 0.880 (95% CI: 0.787-0.972), p=0.030] and testing cohort [0.968(95% CI: 0.942-0.995) vs. 0.863(95% CI: 0.780-0.947), p=0.016], as shown in **Figure 5**.

When radiomics features were added to the PI-RADS, the diagnostic performance of the PI-RADS was significantly improved regardless of whether bpMRI (AUC = 0.982, p= 0.017 for the training cohort and AUC = 0.969, P = 0.007 for the testing cohort) or mpMRI (AUC = 0.986, p= 0.017 for the training cohort and AUC = 0.977, P = 0.008 for the testing cohort) was incorporated.

## Discussion

In this study, we found no significant difference in the diagnostic value of the radiomics model for csPCa regardless of whether bpMRI or mpMRI was incorporated (AUC= 0.975 vs. 0.981, p=0.687 in the training cohort, and AUC= 0.953 vs. 0.968, p=0.287 in the testing cohort). In addition, we demonstrated that the radiomics model outperformed the PI-RADS v2.1 in the diagnosis of csPCa regardless of whether bpMRI (AUC = 0.975 vs. 0.871, p= 0.030 for the training cohort and AUC = 0.953 vs. 0.853, P = 0.024 for the testing cohort) or mpMRI (AUC = 0.981 vs. 0.880, p= 0.030 for the training cohort and AUC = 0.968 vs. 0.863, P = 0.016 for the testing cohort) was incorporated. The addition of quantitative radiomics features to the PI-RADS v2.1 significantly improved the diagnostic value of the combined model for csPCa.

MpMRI has proven to be a robust clinical tool for evaluating the differentiation and aggressiveness evaluation of PCa in patients with a suspicion of PCa. The PI-RADS, as a structured reporting system in prostate MRI that was updated in 2019 to v2.1, has contributed to the success of the technique. The PI-RADS v2.1 simplified the scoring scheme of the mpMRI protocol for reporting and addressed the issue associated with bpMRI (including only T2WI and DWI) for the first time. To date, many studies have investigated the power of the simplified bpMRI protocol, especially in terms of its diagnostic efficiency in PCa. A recent meta-analysis (12) assessed whether the prebiopsy bpMRI could replace mpMRI in the diagnosis of PCa and showed that the pooled specificity demonstrated little difference between bpMRI and mpMRI, but the sensitivity indicated a significant difference. Another two meta-analyses carried out by Alabousi et al. (13) and Woo et al. (14) reached the same conclusions. They both proved that the pooled summary statistics demonstrated no significant difference in sensitivity or specificity between bpMRI and mpMRI. In our study, there were no significant differences between the AUCs of bpMRI and mpMRI (AUCs = 0.871 vs. 0.880, p=0.880 in the training cohort, AUCs = 0.853 vs. 0.863, p= 0.868 in the testing cohort). The sensitivity, specificity and accuracy of bpMRI and mpMRI were 80% vs. 81.7%, 89% vs. 89%, and 85.2% vs. 85.9% in the training cohort and 76% vs. 76%,89.2% vs. 91.9%, and 82.6% vs. 85.5% in the testing cohort. From this point of view, the diagnostic performance of the bpMRI protocol for csPCa is not inferior to that of the mpMRI protocol, and bpMRI has certain advantages in terms of cost reduction, a reduced scanning time, and its non-invasive nature.

However, the role of the DCE sequence in the diagnosis of PCa is rather controversial. In theory, omitting DCE imaging might increase the probability of missing csPCa, as indicated by Greer et al. (15). In their research, the probability of detecting csPCa in PI-RADS 3 + 1 lesions was 54.0%, which was significantly higher than that in all PI-RADS 3 lesions (40.0%). Ullrich et al. (16) found that without DCE imaging, 33% of peripheral higher-grade PCa lesions would be underestimated and misclassified as PI-RADS 3. In our study, the probability of detecting csPCa in positive DCE findings and negative DCE findings were 25% and 20%, respectively, which was lower than previous studies (15, 16). The difference may be related to the diagnostic experience of the radiologists. The total csPCa detection rate was 19.5% and 18.2% in PI-RADS 3 lesions with bpMRI and mpMRI, respectively. Schoots et al. (17) reported an overall csPCa detection rate of 16–21% based on 665 PI-RADS 3 lesions, consistent with our results. Nevertheless, it is still recommended to use DCE imaging as a supplementary sequence for indeterminate lesions, small cancers, lesions in challenging locations and in previously treated prostates (18).

Although the use of the PI-RADS has widened the application value of prostate MRI, it must be noted that the inter-reader variability remains an unsolved problem. To overcome the abovementioned shortcomings, computer-aided approaches have been implemented in recent years to improve diagnosis. Radiomics analysis permits the high-throughput extraction of quantitative features based on machine learning algorithms and has appeared to be a potent tool for the evaluation of characterization of disease patterns. A number of previous studies have outlined the potential of radiomics analysis for PCa diagnosis and risk stratification. Ji et al. (19) developed and validated bp-MRI based radiomics models in two vendors for differentiation between benign and malignant prostate lesions. The bp-MRI radiomics model produced a mean AUC of 0.833, the comprehensive model by combining radiomics model with age and PSA had an improved mean AUC of 0.911. However, their study did not compare the diagnostic value of radiomics based on bpMRI and mpMRI. An earlier review summarized and compared the computer-aided detection and diagnosis of PCa based on mono- and multi-parametric MRI (20). A number of studies have reported that using three modalities leads to better performances than using a mono-modality or a combination of two modalities (21–23). However, in recent years, a few studies have reached different conclusions. Monti et al. (24) compared a standard radiomic model based on T2WI and ADC maps with an advanced model based on DKI and DCE imaging in terms of their diagnostic accuracy for PCa. The radiomics model based on standard features performed better than the predictive model based on advanced features. In our study, we also proved that adding DCE imaging features increased the AUC from 0.975 (95% CI: 0.949-1.000) to 0.981 (95% CI: 0.964-0.999) in the training cohort, and 0.953 (95% CI: 0.920-0.986) to 0.968 (95% CI: 0.942-0.995) in the testing cohort. However, the difference between the diagnostic value of the bpMRI- and mpMRI-based radiomics models was nonsignificant either (p=0.687 in the training cohort and p=0.287 in the testing cohort), which is consistent with Bleker et al. (25) and Cuocolo et al. (26). The addition of DCE imaging to radiomics analysis increased the cumbersomeness of drawing ROIs and the possibility of redundancy in data processing, but its diagnostic efficiency did not significantly improve, thus, it cannot be concluded that machine learning has significantly benefited from the addition of DCE images to the analysis.

Our study also demonstrated that the radiomics model outperformed the PI-RADS v2.1 in the diagnosis of csPCa regardless of whether bpMRI or mpMRI was incorporated. Many studies have proven that the radiomics model is superior to the PI-RADS and clinical indicators in the diagnosis and aggressiveness of PCa (27–30), but few have compared the performance of radiomics and the PI-RADS based on bpMRI and mpMRI. Our research revealed that regardless of whether bpMRI or mpMRI is used, the performance of radiomics is always better than that of the PI-RADS, which further indicates that radiomics can help to improve the diagnostic performance of the PI-RADS v2.1 and is a powerful tool for assisting in the diagnosis of csPCa.

There are several limitations in our study. Firstly, it was a retrospective study performed on data from a single center. Besides, our study did not distinguish PCa that occurred in the PZ and TZ because some highly malignant cases occurred in both zones. The median PSA was relatively high, which also suggest some patients with more advanced disease. For further investigation, a multicenter clinical study with a larger sample size is needed. Additionally, some of the pathological results were obtained from TRUS-guided systematic prostate biopsy. Histological-radiological matching was manually performed by experienced radiologists; thus, the source of bias was inevitable. Therefore, large pathological sections after radical prostatectomy as reference standards and automatic segmentation of lesions are required in the future research. Last but not the least, the main limitation of MRI performance is the fact that it is associated with inter-observer variability. The radiologists for PI-RADS evaluation in our study were well-experienced with more than 5 years of practice, and thus these findings might be better than junior radiologists’ results. In the future, the influence of radiologists with different diagnostic experience on PI-RADS and radiomics analysis will be further studied.

In conclusion, compared with the radiomics model based on bpMRI, the performance of the mpMRI-based radiomics model was not significantly improved, which indicates that omitting DCE imaging in radiomics can simplify the process of analysis. Moreover, the addition of radiomics to the PI-RADS v2.1 has the potential to improve the performance of the structured PI-RADS scheme regardless of whether bpMRI or mpMRI is used, thus enabling us to diagnose csPCa with more confidence.

## Data Availability Statement

The raw data supporting the conclusions of this article will be made available by the authors, without undue reservation.

## Ethics Statement

Written informed consent was not obtained from the individual(s) for the publication of any potentially identifiable images or data included in this article.

## Author Contributions

Conception and design, TC and ST. Administrative support, JS. Provision of study materials or patients, YZ, CW, and WZ. Collection and assembly of data, TC, ST, and SW. Data analysis and interpretation, ZZha, XQ, ZZho, YD, and JH. Manuscript writing, TC. Final approval of manuscript, all authors. All authors contributed to the article and approved the submitted version.

## Funding

The Suzhou Science and Technology Development Plan (Science and Technology Demonstration Project) (Grant No. SS2019012), The Suzhou Science and Technology Development Plan (Basic Research on Medical and Health Applications) (Grant No. SYSD2020113), The Youth Pre-Research Fund of the Second Affiliated Hospital of Soochow University (Grant No. SDFEYQN1814), and The Wuxi Science and Technology Development Plan (NZ2018024).

## Conflict of Interest

The authors declare that the research was conducted in the absence of any commercial or financial relationships that could be construed as a potential conflict of interest.

## Publisher’s Note

All claims expressed in this article are solely those of the authors and do not necessarily represent those of their affiliated organizations, or those of the publisher, the editors and the reviewers. Any product that may be evaluated in this article, or claim that may be made by its manufacturer, is not guaranteed or endorsed by the publisher.
