# Supplementary material for: MRI Based Radiomics Compared With the PI-RADS V2.1 in the Prediction of Clinically Significant Prostate Cancer: Biparametric vs Multiparametric MRI
Source: Front Oncol. 2022 Jan 20;11:792456. doi: 10.3389/fonc.2021.792456 (PMC8810653; doi:10.3389/fonc.2021.792456)
Supplement: Supplementary file 1 [file Table_1.docx]

Supplementary Material

## Supplementary tables

| Variables | | Coefficients |
| --- | --- | --- |
| bpMRI | Intercept | 0.41667 |
| T2_tumor_original_glcm_DifferenceEntropy | | -0.02859 |
| T2_tumor_original_glcm_Imc1 | | -0.05472 |
| T2_tumor_original_glcm_InverseVariance | | -0.02928 |
| ADC1000_tumor_original_shape_Sphericity | | -0.06327 |
| ADC1000_tumor_original_firstorder_10Percentile | | -0.47186 |
| ADC1000_tumor_original_firstorder_90Percentile | | -0.43875 |
| ADC1000_tumor_original_firstorder_Mean | | 0.59821 |
| DC1000_tumor_original_glcm_Imc2 | | -0.07573 |
| ADC1000_tumor_original_glszm_SmallAreaHighGrayLevelEmphasis | | 0.04679 |
| mpMRI | Intercept | 0.41667 |
| T2_tumor_original_shape_Maximum2DDiameterColumn | | -0.04531 |
| T2_tumor_ original_shape_Sphericity | | -0.18547 |
| T2_tumor_ original_glszm_ZonePercentage | | -0.00629 |
| T2_tumor_ original_glszm_ZoneVariance | | 0.28446 |
| ADC1000_tumor_original_shape_SurfaceArea | | -0.11995 |
| ADC1000_ tumor_original_glcm_JointAverage | | -0.04097 |
| ADC1000_tumor_original_ glrlm_GrayLevelNonUniformityNormalized | | 0.09336 |
| ADC1000_ tumor_original_glszm_LargeAreaEmphasis | | -0.62769 |
| ADC1000_ tumor_original_glszm_SmallAreaEmphasis | | 0.106528 |
| Kep_tumor_original_firstorder_TotalEnergy | | 0.55201 |
| Kep_tumor_original_glrlm_GrayLevelVariance | | 0.07490 |
| Ktrans_tumor_original_glrlm_ShortRunLowGrayLevelEmphasis | | 0.10300 |
| Ve_tumor_original_glcm_Imc1 | | -0.05879 |
| Ve_tumor_original_glrlm_ShortRunLowGrayLevelEmphasis | | -0.08245 |
